# Digital chain for pelvic tumor resection with 3D-printed surgical cutting guides

**DOI:** 10.3389/fbioe.2022.991676

**Published:** 2022-09-08

**Authors:** Vincent Biscaccianti, Henri Fragnaud, Jean-Yves Hascoët, Vincent Crenn, Luciano Vidal

**Affiliations:** ^1^ Research Institut in Civil Engineering and Mechanics (GeM), CNRS, UMR 6183, Centrale Nantes, Nantes Université, Nantes, France; ^2^ Department of Orthopedic, Nantes Hospital, CHU Hotel-Dieu, Nantes, France; ^3^ INSERM UMR 1307, CNRS UMR 6075-Team 9 CHILD (Chromatin and Transcriptional Deregulation in Pediatric Bone Sarcoma), Nantes Université, CRCI2NA (Centre de Recherche en Cancérologie et Immunologie Nantes-Angers), Nantes, France

**Keywords:** surgical cutting guides, patient-specific instruments, pelvic bone tumor, reconstruction surgery, 3D printing, additive manufacturing, digital chain

## Abstract

Surgical cutting guides are 3D-printed customized tools that help surgeons during complex surgeries. However, there does not seem to be any set methodology for designing these patient-specific instruments. Recent publications using pelvic surgical guides showed various designs with no clearly classified or standardized features. We, thus, developed a systematic digital chain for processing multimodal medical images (CT and MRI), designing customized surgical cutting guides, and manufacturing them using additive manufacturing. The aim of this study is to describe the steps in the conception of surgical cutting guides used in complex oncological bone tumor pelvic resection. We also analyzed the duration of the surgical cutting guide process and tested its ergonomics and usability with orthopedic surgeons using Sawbones models on simulated tumors. The original digital chain made possible a repeatable design of customized tools in short times. Preliminary testing on synthetic bones showed satisfactory results in terms of design usability. The four artificial tumors (Enneking I, Enneking II, Enneking III, and Enneking I+IV) were successfully resected from the Sawbones model using this digital chain with satisfactory ergonomic outcomes. This work validates a new digital chain conception and production of surgical cutting guides. Further works with quantitative margin assessments on anatomical subjects are needed to better assess the design implications of patient-specific surgical cutting guide instruments in pelvic tumor resections.

## Introduction

Surgical cutting guides (SCGs) are customized tools that help surgeons during complex surgeries (Scolozzi, 2015; Wong et al., 2016). They are patient specific, meaning they are designed and manufactured for a single specific case and tailored to the patient’s anatomy (Vidal et al., 2020a). SCGs are increasingly studied and used in orthopedic and maxillofacial surgeries (Abou-ElFetouh et al., 2011; Krishnan et al., 2012; Wong, 2016). The most common applications are currently for mandibular defect reconstruction and knee surgery. In addition, tumor resection surgeries, orthognathic surgery, or total knee arthroplasty (TKA) also often use this technology (Krishnan et al., 2012; Cartiaux et al., 2014; Gouin et al., 2014; Mazzoni et al., 2015; Greenberg et al., 2021).

There is abundant literature on this topic, and the efficiency of these guides seems to be established (Vidal et al., 2020b). However, there does not seem to be any set methodology for designing such guides (Numajiri et al., 2018). Recent publications, using pelvic surgical guides, showed a wide variety of designs with no clearly classified or standardized features (Wang et al., 2017; Evrard et al., 2019; Siegel et al., 2020; Gkagkalis et al., 2021)**.** The only feature that is common to any surgical guide is the positioning, which uses the negative shape of the bone. García-Sevilla et al. (2021)mentioned that a common strategy was to design wide SCGs to ensure correct placement. The downsides of this strategy are also mentioned (larger SCGs, modification of the surgical procedure to fit the SCG). Another aspect of standardization is the ability to reduce delays (and costs) in the design process. Wong et al. (2015)mentioned a 2-month wait for design and manufacturing. Rustemeyer et al. (2014) mentioned a 2–4-week delay in computer-aided design/computer-aided manufacturing (CAD/CAM) assisted surgeries for maxillofacial applications. In a 2016 review, Martelli et al. (2016) reported that 19.6% of studies using 3D-printed devices found the production delays limiting. We believe that introducing a precise methodology for designing SCGs will improve production times. This methodology should still make extreme customization possible and precisely respond to each case of tumor resection. Pelvic tumor resection SCGs have been documented since 2014 (Cartiaux et al., 2014; Gouin et al., 2014).

The efficiency of SCGs is proven, and the solution they provide might even be preferred to surgical navigation (Wong et al., 2016). However, the descriptions of SCG design digital chains are insufficient, especially for pelvic tumor resections. Numajiri et al. (2018)published a detailed workflow they have developed for patient-specific cutting and reconstruction guides used in fibula free flap maxillary reconstruction. Popescu (2014)described an in-house online platform for the design of SCGs, covering various applications. They highlighted the crucial need for close collaboration and communication between surgeons and designers/engineers to obtain relevant SCGs. They also give a detailed analysis of the numerical workflow, with each step’s input and output file format. Chen et al. (2016)developed a semi-automatic computer-aided method for surgical template design, covering various applications. The method presented focuses on making use of stereolithography (STL) to produce SCGs of various designs by thickening local surfaces directly on the 3D model’s mesh. This then makes it possible to customize the fixation features. However, in-house trials of this approach revealed that direct surface thickening of STL surfaces often failed. Additionally, SCG designs for maxillary resections, long bone resections, or pelvic resections are not similar and need specialized design strategies (Rauch et al., 2021; Vidal et al., 2022).

In this research work, we focused primarily on pelvic bone tumor resections. The aim of our study was to first propose a systematic methodology for designing the SCGs used in pelvic tumor resections. The semi-automatic method we described was assessed in terms of its capacity to produce 3D SCGs, as was the duration of the digital chain process. A complementary experimental approach with the use on a radiopaque synthetic pelvis (Sawbones, Vashon, WA, United States) was performed by trained surgeons.

## Materials and methods

### Study objectives

The aim of this study was to first describe and obtain functional 3D-printed SCGs using our original digital chain methodology, measuring the whole duration of the digital chain process, and evaluating its qualitative efficiency in the hands of specialized orthopedic tumor surgeons on a Sawbones model with regard to ergonomic aspects.

### Systematic digital chain design on patient images

In this study, a systematic workflow (Supplementary Figure S1) was developed to process the DICOM (Digital Imaging and Communication in Medicine) files, design customized SCGs, and manufacture them using additive manufacturing (AM). To carry out the whole process, four types of software were used: 3D Slicer (Fedorov et al., 2012), MeshLab, Siemens NX (Siemens PLM, Plano, TX, United States), and Sinterit Studio. The Segment Editor Extra Effects and Elastix (Klein et al., 2010) of 3D Slicer were also used. The systematic digital chain was first tested on anonymized patients’ images with magnetic resonance imaging (MRI) and computed tomography (CT) sequences.

#### DICOM file processing

To process DICOMs, the 3D Slicer was used. Anatomical 3D reconstruction was obtained by segmenting DICOM image stacks. The chosen approach was multimodal with the use of CT and MRI DICOMs together. To study and test the workflow, archive images of pelvic tumor cases available at the CHU Nantes were studied with full anonymization. The CT images used standard parameters, with a mean slice thickness of 1 mm. The presence of a contrasting agent was not mandatory for selection. MRI images were preferably T1 sequences, with gadolinium contrast agent and a slice thickness between 1 and 2 mm. Other sequences (T2 and STIR) were included if they met the thickness requirements and displayed enough contrast on the tumor. The images were obtained from different machines, such as the GE Medical Systems Optima CT660 (GE Healthcare, Chicago, IL, United States) for CT images, and the Siemens MAGNETOM Sola (Siemens PLM, Plano, TX, United States) for the MRI images.

**FIGURE 1:**
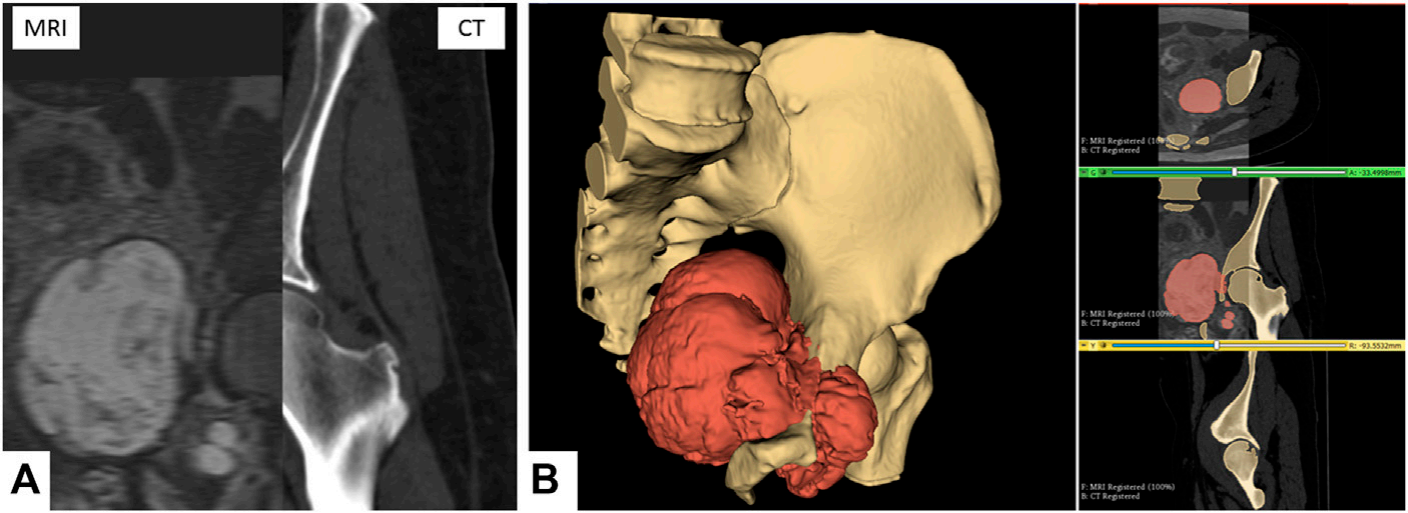
Multimodal segmentation. **(A)** CT and MRI images, non-rigid B-Spline registration (3D Slicer’s Elastix). **(B)** Multimodal segmentation using Watershed (right) and 3D reconstruction (left) of the bone tissues and tumor. Scale = 50 mm.

The multimodal segmentation required prior registration of the DICOM images. CT and MRI were registered using the 3D Slicer’s Elastix module’s B-Spline registration with the “CT/MR-based pseudo-CT (pelvis prostate)” preset (Figure 1A).

One segmentation for each sequence was performed, focusing on the tissue of interest (bone on CT and tumor on MRI). The primary segmentation method used was the Watershed method implemented in the 3D Slicer module Segment Editor Extra Effects (Figure 1B). The reconstructed anatomical 3D models of the bone tissues and tumor were exported in the STL format.

#### STL file processing

**FIGURE 2:**
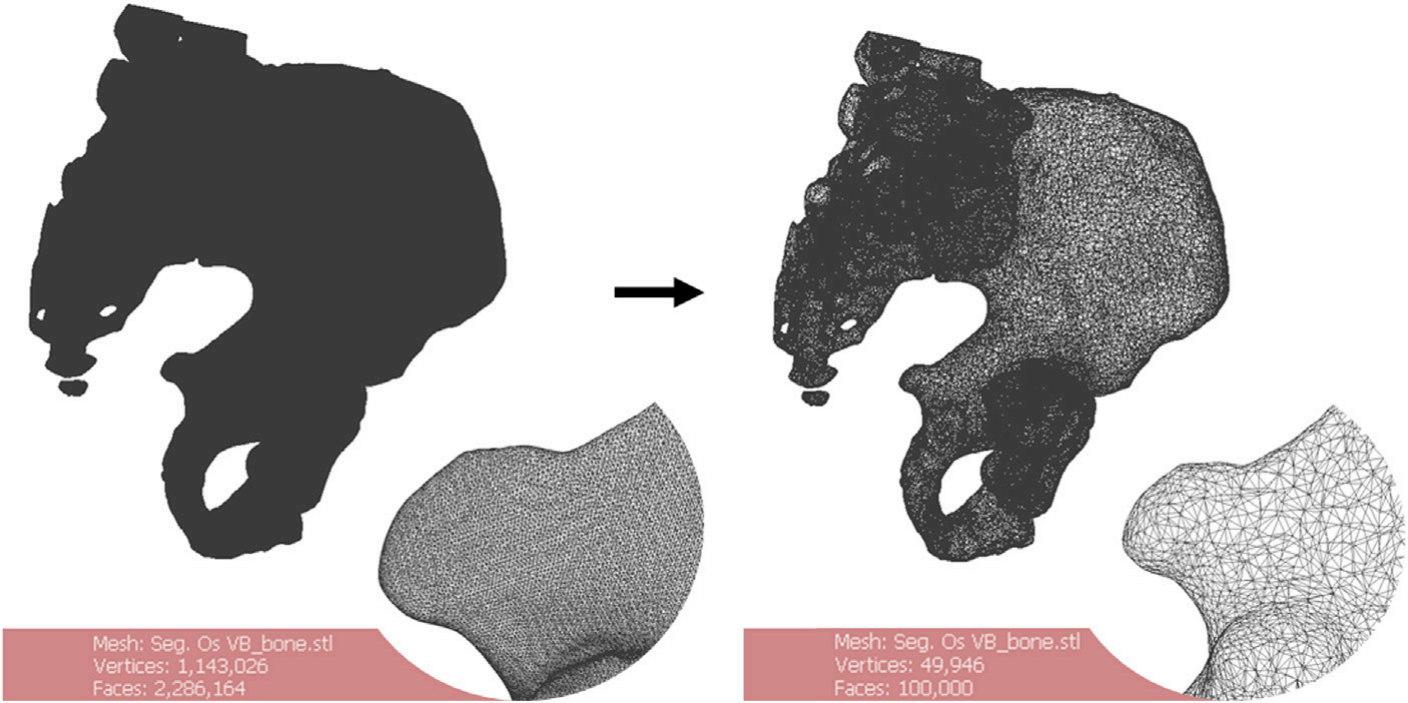
Remeshing of a 3D bone model. The starting number of faces of the mesh (left) is greater than 2.000.000. The oversampling is visible. Remeshing brings the number of faces to 100.000 (right). There is no noticeable quality downgrade for this application. Scale = 50 mm.

The files were remeshed to reduce unnecessary computational load during the computer-aided design (CAD) phase. The quadratic edge collapse decimation function was used to reduce the mesh complexity (Figure 2). Topological differences between the original model and the remeshed model in this case did not exceed 0.68 mm with an absolute mean of 0.01 mm difference (Hausdorff distance computation, sampling on all vertices). The quality downgrade was not noticeable.

#### CAD process and surgical cutting guide design

For the CAD process, Siemens NX was used. The workflow was divided into two main phases: geometrical definition and SCG generation.

**FIGURE 3:**
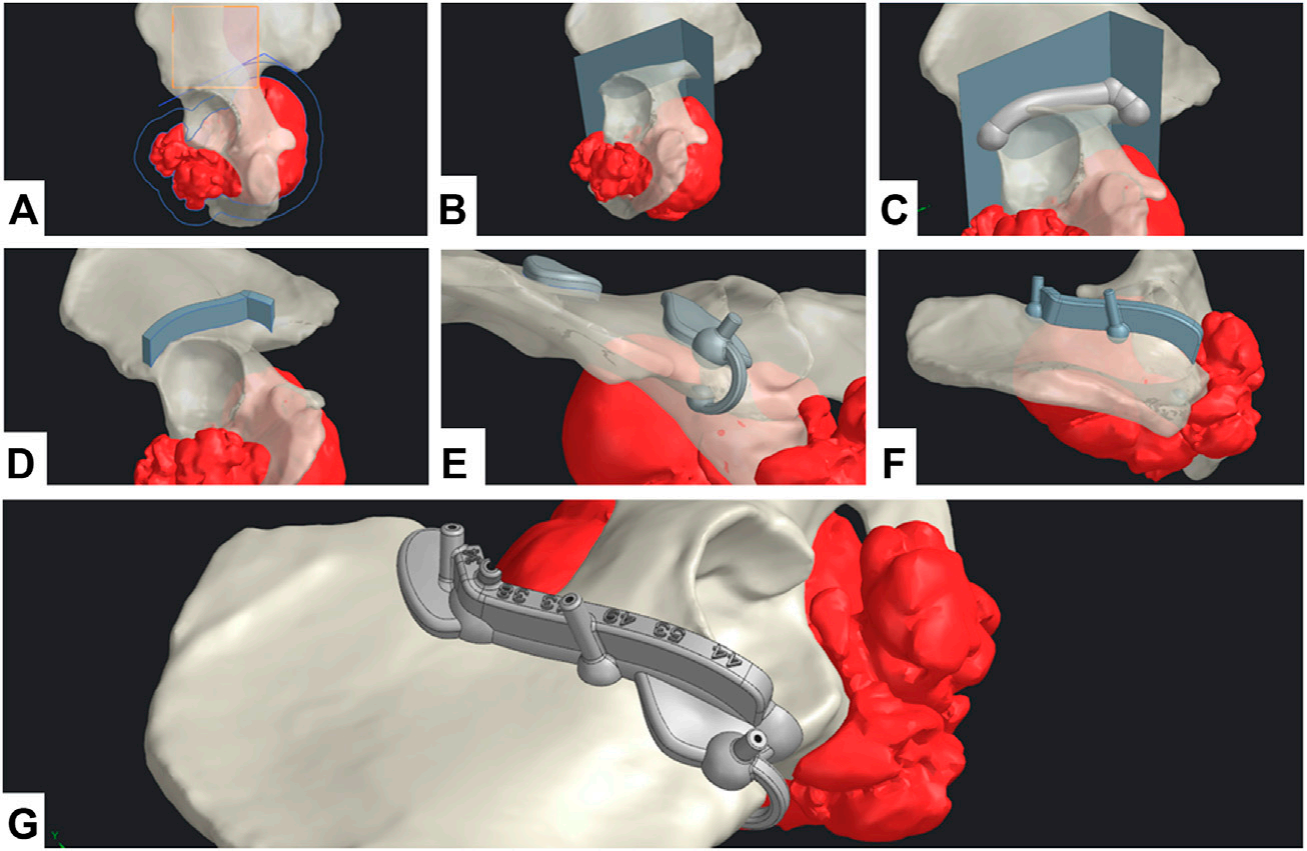
CAD design digital chain. **(A)** Geometrical definition. **(B)** Extrusion of the resection surface. **(C)** Main body generation. **(D)** Guiding feature generation. **(E)** Positioning feature generation. **(F)** Fixation feature generation. **(G)** Final SCG with united bodies and finishing features.

A straight collaboration between clinicians and engineers was established for the geometrical definition phase. A 3-point plane was defined, representing the main surgical direction of the approach. A polyline sketch on this plane defined the broad trajectory of resection (Figure 3A). The tumor’s silhouette was projected on to the sketch and expanded by a 12 mm margin to conserve the oncological surgical margin of resection. The polyline was extruded as a surface body with a 5° draft angle (resection surface, Figure 3B). The SCG was generated in five steps: main body, guiding feature, positioning features, fixation features, and finishing. A 3D-trajectory was created along the resection surface for the main body: the intersection curve between the resection surface and bone model was displayed. Then, two extremities were defined for the cut. The intersection curve was simplified by approximating it to a 3D-spline between the nodes (nodes = extremity or intersection point between the bone model and resection plane junctions). The main body was generated as a solid circular sweep of diameter 16 mm along the 3D trajectory. The segments of the body were linked by spherical anchors (Ø20 mm) at each node (Figure 3C). The 3D trajectory was offset from 15 mm upward along the resection surface. This defined the upper limit of the guiding feature. The surface was then cut using both 3D trajectories (initial and offset) to create the inner guiding surface. This surface was thickened by 7 mm to create the guiding feature’s solid body (Figure 3D). The blade and pin axis were designed to avoid hurting any critical anatomical structures.

For the positioning features, two options were available: local contact surface widening (CSW) and deformable clip. A thin surface adherent to the bone in the area was thickened by 5 mm to create an additional contact surface. The width of the CSW could be limited to maximum defined by the surgeon. An offset copy of the cutting surface was used as a limiting element for the cutting guide’s width. A Boolean subtraction was made to remove all parts of the SCGs outside the defined limits. These positioning features should not bring any risk or unnecessary exposure to noble anatomical structures. Finally, if the edge of the bone at the extremity was thin enough, a deformable clip was added. Two spheres were positioned on each side of the edge and linked with a thin bridge. Both local CSW and deformable clips could be used simultaneously (Figure 3E). The fixation features were designed as a drilled cylinder (Ø6 mm exterior, Ø2.1 mm interior) with a spherical anchor. Fixation feature axes were contained in the respective outer planes of the guiding feature. The axes were tilted from the direction of approach, so that none would be parallel with any other (Figure 3F). Finally, finishing was done by uniting all the solid bodies, and then creating corner gaps and cutting depth indications (Figure 3G).

#### Additive manufacturing

For the additive manufacturing process, the selective laser sintering (SLS) technology was chosen. The SCG prototypes were additively manufactured using a Sinterit Lisa SLS 3D (Sinterit, Krakow, Poland) printer with the affiliated software, Sinterit Studio. The material chosen was a nylon polyamide, Sinterit Polyamide 12 (PA12).

### Evaluation method on a radiopaque synthetic pelvis

#### Experimental conditions

##### Bone tumor simulation

**FIGURE 4:**
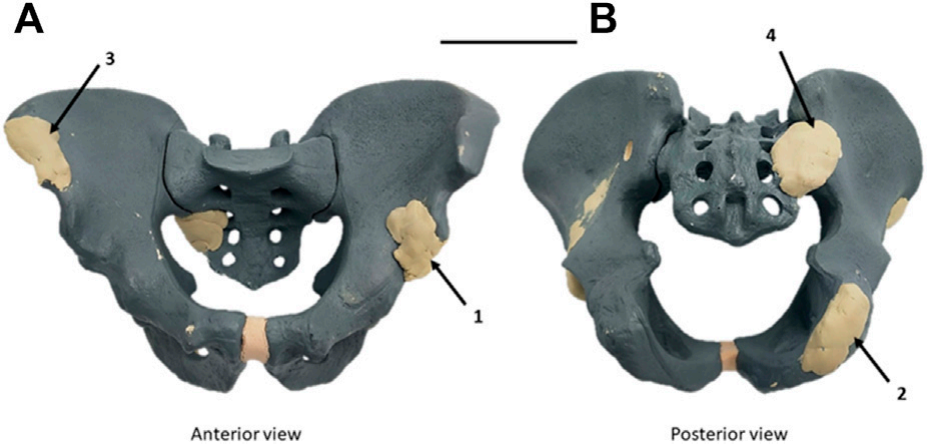
Radiopaque synthetic Sawbones model of the pelvic ring with artificial epoxy tumors. **(A)** Antero-superior view. **(B)** Postero-inferior view. Four tumors were implanted: left acetabulum (Zone II Enneking) (1), right ischium (Zone III Enneking) (2), right iliac crest (Zone I Enneking) (3), and right sacroiliac junction (zone I+IV Enneking) (4) (Enneking et al., 1980). Scale = 100 mm.

The experimental study was performed to validate our design digital chain. In a radiopaque synthetic pelvis (Sawbones, Vashon, WA, United States), four artificial epoxy tumors were developed: right acetabulum (Enneking Zone II), left ischium (Enneking zone III), left iliac crest (Enneking zone I), and left sacroiliac junction (Enneking zone I+IV) as shown in Figure 4 (Enneking et al., 1980).

A CT image was then acquired on the modified synthetic pelvis, using the Philips Ingenuity Flex scanner (Philips International B.V., Amsterdam, Netherlands). Unlike the first method described previously for patient images, and given that no MRI sequence was acquired as dry synthetic bones are not MRI compatible, only a mono-modal (CT only) segmentation was thus performed in the 3D Slicer. Watershed segmentation was used as described. The contrast on artificial tumors was enough to clearly distinguish them from the synthetic cancellous bone. Some minor uncertainties remained on cortical bone (similar Hounsfield Units), requiring corrections in the first Watershed iteration. The 3D model was then exported in the STL format and remeshed. The STL was imported into Siemens NX for the CAD design of each SCG. The duration of the digital chain process was assessed for each simulated tumor.

##### Margins and cut planning

Surgical margins with a 10 mm margin baseline and local reductions to 5 mm for bone preservation considerations were defined by the surgeons. They helped establish baseline parameters for the CAD design (geometrical definition). On each case, a close clinician/engineer collaboration was needed to determine the correct surgical approach and the number of resection planes.

#### Resection protocol

Three surgeons (two senior specialized surgeons and one junior surgeon) participated in this study. Using our design digital chain, SCGs were produced for the four pelvic tumor resection cases. Iterations were made to test out different positioning features and different resection strategies. In total, the digital chain was used 12 times on the synthetic pelvis. All resections were carried out on the same day by the same team of surgeons, using the same process. The SCGs were fixed to the synthetic bone with Ø2mm K-Wires.

The resections were carried out with a Stryker^™^ motor and a 90 × 13 × 1.27 mm oscillating sawblade. A Sawbones model was fixed to a universal Sawbones clamp which can swivel 360° and rotate to any position, vertical or horizontal, aiming to reproduce patient positioning during the conventional surgery. During and after surgery simulation, the clinician was asked to evaluate the quality of the SCG’s positioning, as well as its stability.

#### Qualitative assessment

The evaluation was made based on the subjective feelings of the surgeon: was the correct position of the SCG easy to find and maintain? (Primary positioning: Very easy/Easy/Medium/Hard/Very Hard/Impossible). Was the SCG stable prior to fixation? (Primary stability: Excellent/Good/Medium/Unstable). Was the SCG stable after fixation? (Secondary stability: Excellent/Good/Medium/Unstable). Was the SCG stable during resection? (Stability during the cut: Excellent/Good/Medium/Unstable). Was the resected piece easy to remove? (Extraction of resected piece: Easy/Medium/Complex/Impossible). The presence of bone fractures was also assessed (Yes/No). Finally, on a side note, the damage sustained by the SCG were also evaluated: did the guide sustain significant damage? (Overall integrity: No damage/Non-critical damage/Critical damage) Were there particle deposits due to the sawblade’s friction with the SCG? (Particle deposits: No deposits/Small deposits/Significant deposits). The time taken by the surgeon to accomplish the various phases was also measured.

## Results

### Evaluating digital chain production and assessing duration

The digital chain could be used in every case and produced the expected results. No major glitch in the workflow was experienced, with the main difficulty being local holes in bone shape on the path of the SCG. This happened in two distinct anatomical areas: in the sacroiliac area (sacral foramen) and the ischiopubic ramus (obturator foramen).

The variety of cases made it possible to test SCGs with one–four resection planes. A first fit-test was a simple positioning of the guides on the dry bone around the simulated tumors. This made it possible to test the primary positioning for all 12 iterations. In total, two SCGs presented one resection plane, six SCGs presented two resection planes, two SCGs presented three resection planes, and two SCGs presented four resection planes. SCGs with three resection planes or more were easier to position and to maintain in place before K-Wire fixation. SCGs with two resection planes were slightly less stable and thus needed good positioning features. SCGs with one resection plane were difficult to position, significantly less stable, and were thus not used for the cutting test. The most convincing version for each SCG was then selected to perform the resection.

### Evaluation on a radiopaque synthetic pelvis

**FIGURE 5:**
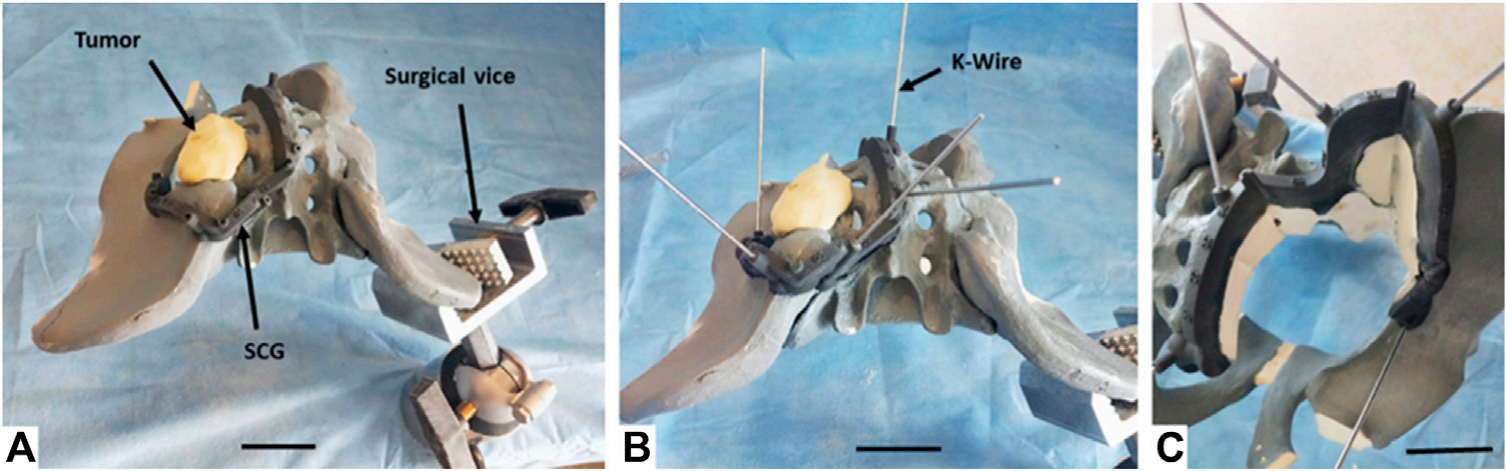
Resection process performed by the surgeon on the left sacroiliac junction tumor (zone I+IV Enneking) on the Sawbones model. **(A)** Primary positioning of the SCG. **(B)** Fixation of the SCG using nonparallel K-Wires. **(C)** Resection of the artificial tumor and extraction of the resected piece. The last phase (not in figure) consisted in removing the K-Wires and the SCG. Note: The synthetic pelvis was positioned in an orientable surgical vice. For each resection, the pelvis was oriented to simulate the patient’s position in a realistic case. The areas where the patient’s body would be located were not used by the surgeon.

Using selected SCGs from the previous experimental step, the four artificial tumors implanted in the radiopaque Sawbones model (Sawbones, Vashon, WA, United States) were successfully resected using the digital chain (Figure 5). No traces of epoxy were found on the resection planes. During and after the simulated surgery, the clinician was asked to evaluate the quality of positioning the SCG, as well as its stability and ergonomics, with mostly very good and excellent results (Table 1).

**TABLE 1:** Evaluation of the four artificial tumor resections.

Criteria	Evaluation modality	Tumor 1 (acetabulum)	Tumor 2 (ischium)	Tumor 3 (iliac crest)	Tumor4 (sacroiliac junction)
Enneking zone	Enneking classification (Enneking et al., 1980)	II	III	I	I + IV
Number of cutting planes	—	3	2	2	4
Epoxy traces on cutting planes?	Yes/no	No	No	No	No
Number of positioning trials	—	1	1	1	2
Primary positioning (without fixation)	Very easy/easy/medium/hard/very hard/impossible	Very easy	Easy	Very easy	Medium
Primary stability (without fixation)	Excellent/good/medium/unstable	Good	Medium	Good	Excellent
Secondary stability (with fixation)	Excellent/good/medium/unstable	Excellent	Excellent	Excellent	Excellent
Stability during the cut	Excellent/good/medium/unstable	Excellent	Excellent	Excellent	Excellent
Extraction of resected piece	Easy/medium/complex/impossible	Easy	Easy	Easy	Medium
Fracture	Yes/no	No	No	No	No
Overall integrity of SCG	No damage/non-critical damage/critical damage	No damage	No damage	No damage	No damage
Particle deposits	No deposits/small deposits/significant deposits	Small deposits	Significant deposits	Small deposits	Significant deposits

The surgeon evaluated the SCG during the resections.

Installing the SCG took between 60 and 92 s (primary positioning + fixation) (Table 2). The exact surgical margin achieved was not evaluated, but as there was no resin on the Sawbones cut, we can assume that it was a least a “macroscopically complete” resection.

**TABLE 2:** Duration measurements (seconds) for each phase of use of the SCG.

Time (s)	Tumor 1 (acetabulum)	Tumor 2 (ischium)	Tumor 3 (iliac crest)	Tumor 4 (sacroiliac junction)
Primary positioning (without fixation)	7	6	10	21
Fixation (K-Wire installation)	85	54	60	59
Cut	157	41	68	240
Extraction of resected piece	0 (fall)	0 (fall)	0 (fall)	2
SCG dismounting	23	15	20	24
Total	272	116	158	346

The mention “fall” indicates that the resected piece fell on its own when the surgeon completed the resection.

The inclined resection planes proved to be efficient in easing the extraction of the resected piece (the resected piece either fell out directly or took a few seconds to extract (Table 2)). No fracture was observed with the Sawbones specimen piece removal. Finally, the SCGs sustained the cutting efforts with no critical damage. However, some particle deposits were observed, in variable amounts. These deposits were due to the oscillating blade, superficially damaging the cutting guide during the resection. Minor misalignments of the blade on the cutting plane by the operator and vibrations of the blade caused this damage. The damage translated into small particles of PA12 being ripped from the guide and deposited on the cutting site. The amount of PA12 deposited could not be accurately determined as the particles were mixed with Sawbones dust. However, most of the damaged parts remained attached to the guide because of PA12’s ductility.

## Discussion

The efficiency of 3D-printed surgical cutting guides seems proven in the recent literature (Sallent et al., 2017; Wang et al., 2017; Bosma et al., 2018). However, there is a lack of description of design digital chains for these guides. Maxillofacial reconstruction seems to be the most prominent field for developing SCG design workflows (Numajiri et al., 2018; Ostas et al., 2021), but it remains rare. To the best of our knowledge, such workflows do not exist for pelvic applications in the literature, especially for tumor resection surgeries.

### Literature review concerning pelvic tumor resection SCGs

Different articles making use of pelvic SCGs (Table 3) were analyzed to build the digital chain described in our study in material and methods. The analyzed literature included articles about pelvic tumor resection (with reconstruction or not) that showed images of the SCG. Sawbones, cadaveric, and clinical studies were included. We also included two studies on the cut precision for pelvic osteotomy guides (Sallent et al., 2017; García-Sevilla et al., 2021). These articles did not display a tumor resection situation, with its complexity, but were still valuable for understanding SCG placement on pelvic bones. The examples of SCGs for all four zones of the pelvis (Enneking classification, F. Enneking et al., 1980) were found. A total of 13 publications were identified.

**TABLE 3:** Literature analysis for pelvic tumor resection guide designs.

Author reference	Date	Enneking zone	Number of resection planes per SCG	Detailed design workflow	Design software	Types of blade guiding	General thickness	Positioning method	Fixation method	Additional features	Manufacturing process	Material
M. García-Sevilla et al. (García-Sevilla et al., 2021)	Mar-21	I, III	1	Yes	Meshmixer	Flat open border	1	Negative cortical bone shape + global contact surface widening	Screws		LFS	Dental SG resin
K.-C. Wong et al. (Wong et al., 2016)	Feb-16	I, II	3	Yes	Magics RP (Materialize)	Elevated closed slit	4	Negative cortical bone shape + global contact surface widening + stretched positioners	K-Wires		FDM	ABS
F. Gouin et al. (Gouin et al., 2014)	Jul-14	II, IV	1–4	No	Blender	Elevated open border	1 SCG = 2, 1 SCG = 3, 1 SCG = 5	Negative cortical bone shape + global contact surface widening	K-Wires	Metal sleeves for K-Wires	SLS	PA12
A. Sallent et al. (Sallent et al., 2017)	Oct-17	I, II, III, IV	1–2	No	3Matic (Materialise)	Elevated open border	2	Negative cortical bone shape + global contact surface widening	K-Wires		SLS	PA12
O. Cartiaux et al. (Cartiaux et al., 2014)	Jan-14	I, III	1–2	Yes (rough)	In-house software	Elevated open border	5	Negative cortical bone shape + global contact surface widening	K-Wires		SLS	PA12
K. C. Wong et al. (Wong et al., 2015)	Jan-15	II, III	1–3	Yes	3Matic (Materialise)	Elevated open slit	3	Negative cortical bone shape + global contact surface widening	K-Wires		SLS	PA12
T. Jentzsch et al. (Jentzsch et al., 2016)	Dec-16	I	2	No	CASPA	Elevated open border	5	Negative cortical bone shape + global contact surface widening	Not mentioned or visible		SLS	PA12
R. Evrard et al. (Evrard et al., 2019)	Jun-19	I, II, IV	1	Yes (rough)	Outsourced to 3D-Side, Leuven, Belgium	Elevated open border	2	Negative cortical bone shape + local contact surface widening + stretched positioners	K-Wires		SLS	PA12
G. Gkagkalis et al. (Gkagkalis et al., 2021)	Apr-21	II, III	1–2	No	Outsourced to Materialise	Elevated closed slit	3	Negative cortical bone shape + global contact surface widening	K-Wires		Unknown	Unknown
M. A. Siegel et al. (Siegel et al., 2020)	Nov-20	IV	3	No	Outsourced to BodyCad, Montreal, Canada	Elevated closed slit	3	Negative cortical bone shape + local contact surface widening	Unknown	Series of drill guides	SLS	PA12
E. Cernat et al. (Cernat et al., 2016)	Oct-16	I, IV	2–4	No	Unknown	Elevated open border	3	Negative cortical bone shape + local contact surface widening	K-Wires		SLS	PA12
B. Wang et al. (Wang et al., 2017)	Mar-18	I	2	No	Unknown	Flat open border	1	Negative cortical bone shape + global contact surface widening + stretched positioners	K-Wires		Unknown	Unknown
X. Fang et al. (Fang et al., 2018)	Dec-18	I, II, III, IV	2	No	Creo 2.0 (PTC, Needham, MA, United States)	Elevated open border	3	Negative cortical bone shape	K-Wires		Unknown	Unknown
Z. Yu et al. (Yu et al., 2021)	Apr-21	I, IV	2	No	Creo 2.0 (PTC, Needham, MA, United States)	Elevated closed slit		Negative cortical bone shape + local contact surface widening	K-Wires	Fixation on both sides of cutting planes	Unknown	Unknown

The information is based on article contents. The general thickness was estimated visually (1 = very thin/2 = thin/3 = medium/4 = thick/5 = very thick). The main positioning features were also considered. The negative cortical bone shape feature was common to every guide. 10 guides used global contact surface widening (wide guides). Three guides used local widening (thin guides overall with wider local features). Three guides additionally used stretched positioners (features distant from the resection planes, supposedly to stabilize the guide and use specific bone landmarks). Most guides used K-Wire fixation and SLS additive manufacturing in PA12 material. FDM, Fused deposition modelling; SLS, selective laser sintering; and LFS, low-force stereolithography.

#### SCG workflow

The complexity of the resection also varied between one and four cutting planes. The analysis was based on workflow explanations in the articles (Wong et al., 2016; García-Sevilla et al., 2021) or visual assessments (Cernat et al., 2016; Jentzsch et al., 2016; Gkagkalis et al., 2021). It showed that the design workflow of SCGs for pelvic tumor resections has rarely been detailed, justifying our methodology-based original article.

#### SCG designs

Concerning the SCG design, we highlighted high heterogeneity in designs in the literature. Nonetheless, elevated open border guiding appeared to be the most common guiding feature in the batch (6 out of 13). It was not possible to precisely determine the height of the guiding surfaces due to the lack of information in the Materials and methods sections in these publications. The more recent studies display thinner cutting guides. The baseline feature for positioning was always that the SCG was designed as a negative shape of the cortical bone. However, the evaluation of the design of the contact surface was also made: in most cases (10 out of 13), the contact surface was globally wide, along the entire length of the guide. This observation was also made by García-Sevilla et al. (García-Sevilla et al., 2021). Two examples showed very thin guides with widening in local areas. Three cases showed SCGs with additional stretched positioners. These features provide an additional contact area far from the cutting planes. In 10 out of 13 cases, fixation was made with the Kirschner wires (K-Wires). Finally, eight out of 13 SCG examples used selective laser sintering (SLS) with nylon (PA12) for the manufacturing process.

### Evaluating digital chain production

#### 3D processing steps

##### DICOM processing phase

For the DICOM processing phase, the 3D Slicer (Fedorov et al., 2012) was selected. As it is open-source and customizable, an in-house Slicelet (3D Slicer module) that translated the DICOM processing part of the digital chain was built. To develop this part, we chose CT and MRI as the main image modalities. In the orthopedic field, CT is the gold standard for bone tissue segmentation. In the oncologic field, MRI is preferred for tumor segmentation. From these gold standards, our approach was to propose multimodal segmentation. 3D bone tissue models are reconstructed using CT images, and tumor models are reconstructed using MRI images. CT images are usually naturally suited for segmentation (high resolution, low slice thickness, and good bone contrast). The range for MRI settings is wider. T1 sequences using gadolinium as the contrast agent were the most efficient for highlighting the tumor well. The slice thickness should be between 1 and 2 mm for easier registration and semi-automatic segmentation. Using thicker MRI (4- or 5-mm slice thickness) is also possible, but less adapted to semi-automatic segmentation methods. The multimodal registration was performed using B-Spline registration. We found Elastix to be the best performing automatic registration module in 3D Slicer (Figure 1A) (Klein et al., 2010) for pelvic images and chose to use it in the study. We also found the Watershed method to be an excellent segmentation method for this digital chain: the manual part (initialization) was fast and simple and did not require high user accuracy. The calculation then expanded the initialized segments automatically, creating plain and smooth 3D models, natively suited to CAD processing (Figure 1B). The major downside of the Watershed model was the intensive calculation. We also noted that Watershed performed poorly on hollow structures, such as the skull or structures with numerous small details/ramifications, such as the brain’s grey matter or small vascularization. It produced very good performances on blocky or clearly defined features, such as pelvic bones on CT or tumors on MRI. It could also efficiently segment the main vascularization system (aorta, major arteries, and veins) if a contrast agent was used in the CT or MRI acquisition. The efficiency of Watershed segmentation has been shown in the literature for automatic and semi-automatic segmentation processes. It is said to perform adequate segmentation while saving time (Fan et al., 2019). The segmentation process could be improved by automating the initialization part. A hybrid thresholding approach is presented by Kim et al. (Kim et al., 2018) for an acetabulum automated segmentation process.

##### STL processing phase

For STL processing, MeshLab (Cignoni et al., 2008) was used for its easily accessible remeshing functions (Figure 2). STL files exported from 3D Slicer were in general very heavy. This was due to the extra fine default settings used by 3D Slicer on complex models. Such heavy meshes are translated into computational stress on the CAD software. The process is fully automatable, providing fixed size requirements for the STL (target number of faces or file weight for example) (Figure 2).

##### CAD processing phase

For the CAD phase, Siemens NX (Siemens PLM, Plano, TX, United States) was used. The lattice structure generation capabilities of the software were also considered very profitable, especially from the perspective of integrating a reconstruction phase into the digital chain. During this study, an engineer performed the geometrical definition with the information provided by surgeons. However, the goal in this digital chain would be to have the geometrical definition performed directly by clinicians to obtain optimal translation of surgical planning in the CAD 3D space. This phase aims at translating surgical constraints and objectives into geometrical objects. These objects are then used as references to generate solid bodies. The essential surgical parameters, such as the surgical margins, were defined with an orthopedic surgeon and included the blade thickness (10 mm margin + 2 mm thick blade). An interactive planning between the engineer and surgeon is mandatory not only for the surgical margins but also for cutting plane choices and K-Wire localization to avoid hurting noble anatomical structures with the saw or pins (vessels, nerves, bowels, and bladder).

#### SCG generation

The steps for generating SCG were performed manually by an engineer following a systematic method. However, provided there is development of an in-house platform, most of the steps could be automated.

##### SCG design choices

Most design choices were based on the analysis of the literature: the main body was made as a cylindrical 3D sweep to ensure continuous contact with the cortical bone in all situations. An extrusion-based design (Wong et al., 2016) can be an efficient technique, but we found that high topological variations and resection complexity are detrimental to the repeatability of this method. We also acknowledged that most cutting guides are designed with a wide contact surface on the whole cutting length (Cartiaux et al., 2014; Gouin et al., 2014; Wong et al., 2015; Jentzsch et al., 2016; Sallent et al., 2017). However, to avoid additional trauma, we chose to design thin guides. For this reason, the main body of the SCG is only up to 8 mm wide (half of the cylindrical sweep diameter). Nevertheless, the main body alone was not enough to correctly position the SCG. Therefore, added positioning features were added that were similar to what Siegel et al. (2020), Evrard et al. (2019), and Cernat et al. (2016) recommended. These positioning features were added preferably in accessible areas of the bone to limit additional dissection, and their width can be limited to a maximum defined by the surgeon. The deformable clips are a test feature aimed at improving the first positioning of the guide and stabilizing it before K-Wire fixation. The 5 mm thick bridge is easily deformable to pinch the bone’s edge and maintain the guide. It is unclear if this feature would be beneficial in actual surgery. The 15 mm guiding height was decided in collaboration with the experienced surgeons. We also confirmed it visually in the literature, as Sallent et al. used a height of 20 mm, although in other cases the guiding height is rarely specified. The 7 mm thickness was also a parameter defined by surgeons to avoid unnecessary dissection while withstanding the effort and oscillations of the blade. On this specific thickness point, the literature identifies two global conceptions: wide thickness SCGs and thinner ones.

We also added corner clearances that prevent the blade from damaging the guide at the intersections of resection planes, as described in the literature (Gouin et al., 2014). We found it impractical to implement a physical limitation on the depth of the blade. Displaying depth information to help the surgeon seemed a better option. Nonetheless, any extensive dissection toward noble structures (nerve roots and iliac vessels) was avoided. The blade and pin axes were also designed to avoid these critical anatomical areas if the tumor resection strategy did not plan for their resection.

##### SCG production

For additive manufacturing, SLS printing with PA12 is a certified process and material in the medical field. SLS is a powder-based additive manufacturing technology. It makes it possible to print complex objects in a powder volume. The powder is sintered layer by layer using a laser. This technology supports various materials from polymers (PA12 and TPE) to metals (steel and titanium). In this study, we chose polymer SLS printing for its ability to print complex free-form objects without using a support material (unlike fused deposition modeling (FDM)). It produced accurate printing of the SCGs. It is also the most popular choice in pelvic resection guides in the literature (Cartiaux et al., 2014; Gouin et al., 2014; Wong et al., 2015; Jentzsch et al., 2016; Sallent et al., 2017; Evrard et al., 2019; Siegel et al., 2020). PA12 was the material selected for this study. It is a common material that is easy to work with in SLS printing, and its manufacturing parameters are perfectly well-known for the Sinterit Lisa machine. It is also biocompatible and suited to AutoClave sterilization. PA12 was thus indicated to produce the SCGs used in this study. SLA printing with Dental SG resin (or a similar material) could also be a viable option, as shown by García-Sevilla et al. (2021).

### SCG and duration assessment

Using our semi-automated method, the digital chain succeeded in producing one SCG in less than 20 h, without taking into account the duration of the CT imaging procedure for the pelvic Sawbones. The longest step in the process was the printing of the SCG itself (11 h for one SCG), making printing batches for multiple SCGs might critically reduce the duration of this step (46 h for seven SCG). It should be noted that automating several CAD steps (which were designed to ultimately be automated) will reduce the duration of the SCG design in the near future.

The 20-h process, we report, to produce a single SCG does not take into account all the waiting time between each step, which may have biased our result. Moreover, discussion between the clinician and conception team might slow down the process. It is mandatory that feedback be obtained quickly from the surgeons and engineers to reduce the duration of the SCG process. This might be of major importance in an acute emergency oncological context and in trauma case situations, which might become an ongoing field for patient-specific tools in a reconstructive context.

Nonetheless, our results seem critical as most production times in the literature are of more than 2 weeks (Rustemeyer, 2014; Martelli et al., 2016). We believe that the semi-automated methodology we describe here could reduce production times for the patient-specific SCG instruments.

### Evaluating a radiopaque synthetic pelvis

The surgically simulated evaluation with experienced bone tumor surgeons highlights good ergonomics and repeatability on Sawbones models. Interestingly, the easier-to-fit SCG were those with more than two planes in our experiment. The one-plane SCGs were rapidly left aside due to a lack of initial stability, meaning that this one-plane design might need complementary cross-sectional supports, if mandatory. An overall ergonomic assessment by surgeons was very good and excellent for most parameters. No resection piece fracture was identified, and nor was there any critical damage to the SCGs. These results transposed to human cases are nonetheless very debatable, due to the easy access of the Sawbones on a clamp, and to the lack of any soft tissue that might complicate exposure and SCG placement. Nonetheless, the aim of this study with this Sawbones evaluation remains a qualitative assessment, and at this point, the results seem positive, with fast and easy use of the generated SCGs . The four artificial tumors were successfully resected from the Sawbones model using this digital chain. This provided the first confirmation of the usability of the SCGs generated, as they showed repeatable results both in terms of their designs and their performance.

### Limits and strengths

In this preliminary methodological original publication, we do not assess margin quality with precise CT measurements. Nonetheless, no traces of epoxy were found on the cut evaluations, and we can consider this to be a “macroscopically complete” resection. This work mainly focused on the SCG design, a description of digital chain production, and quality assessment with qualitative and ergonomic data. The surgeon’s qualitative analysis of the SCG’s strength during its use seemed satisfying, but no quantitative force analysis has been performed. Such analysis would require the evaluation of the effort applied by the operator on the SCG with the oscillating saw, and specific methodology on this point might be developed for better assessing the SCG strength. Given that this is an experimental pilot study to validate our digital chain, no statistical analysis was performed.

Further study with greater sample size and quantitative assessment is needed to compare the design impact on scheduled margins and to validate this pilot study. This next step experiment will focus on Sawbones models and anatomical subjects, in order to take into account the importance of “real surgery” situations, such as patient soft tissue with surgical exposure difficulties and more constraints against the SCG. We may assume that with anatomical subjects, resection times might be much longer that the one we identified in our Sawbones qualitative assessment, with more pitfalls. Extending the images used in our digital chain to 3DMMI (3D multimodality image) that include CTA (CT angiography) and MRN (Magnetic Resonance Neurography) would be valuable to include the tumor adjacent noble vital structures in the surgical planning phase 3D models. (Fang et al., 2018; Yu et al., 2021).

Finally, our multimodal workflow brings new insight. If there are publications focusing on combined MRI and CT use for SCG design purposes (Table 3), to the best of our knowledge, documented and repeatable pipeline designs have not been fully documented, especially for pelvic resections.

### Conclusion

In this study, we developed an original and fully detailed new design digital chain for pelvic tumor resection surgical guides. The digital chain covers multimodal DICOM segmentation, STL processing, CAD design, and additive manufacturing. It was developed in an in-depth surgical collaboration context. This digital chain could be adapted to different clinical specialties and extended to additional applications outside the bone tumor field.

SCG production was considered successful on Sawbones experiments, and its surgical simulation was satisfactory in terms of ergonomics. Four artificial tumors were successfully resected from a Sawbones model using the digital chain. The design and production delays were also satisfying. The multimodal workflow could have been validated through this experimental study. Cadaveric and clinical case studies are scheduled to confirm these results with a quantitative assessment of various SCG designs.

## Data Availability

The raw data supporting the conclusions of this article will be made available by the authors, without undue reservation.
