# Supplementary figures and images for: Digital chain for pelvic tumor resection with 3D-printed surgical cutting guides
Source: Front Bioeng Biotechnol. 2022 Sep 8;10:991676. doi: 10.3389/fbioe.2022.991676 (PMC9493251; doi:10.3389/fbioe.2022.991676)

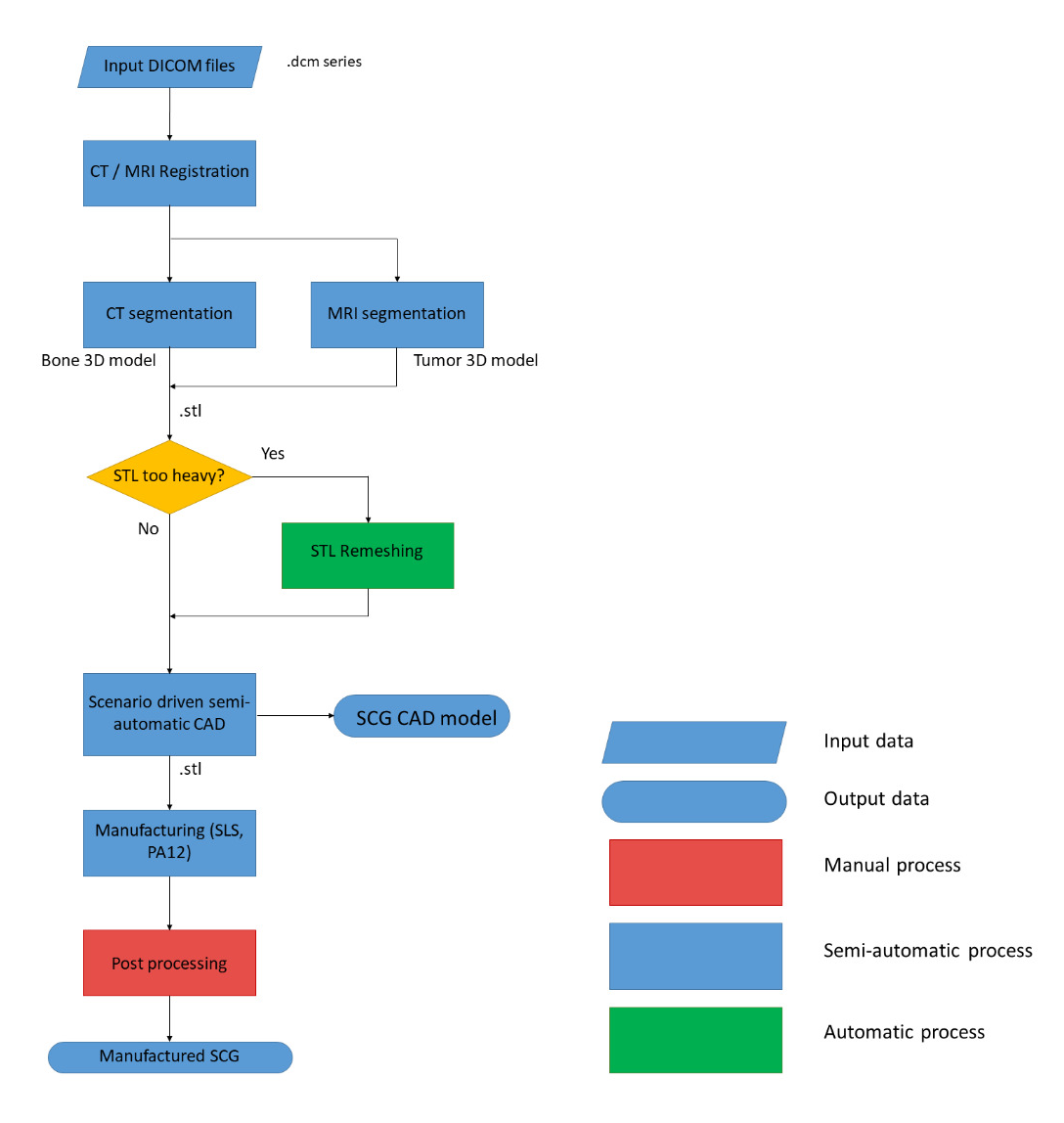

Supplement: Supplementary file 1 [file Image1.PNG]
